# Sociodemographic differences in dementia prevention knowledge in Germany: Implications for targeted health communication

**DOI:** 10.1016/j.tjpad.2026.100517

**Published:** 2026-02-28

**Authors:** Pauline Albus, Ann-Kristin Folkerts, Josef Kessler, Sebastian Köhler, Elke Kalbe

**Affiliations:** a Medical Psychology | Neuropsychology and Gender Studies & Center for Neuropsychological Diagnostics and Intervention, Faculty of Medicine and University Hospital Cologne, University of Cologne, Cologne, Germany; b Alzheimer Centrum Limburg, Mental Health and Neuroscience Research Institute, Maastricht University Medical Center+, Maastricht, the Netherlands; c Department of Psychiatry and Neuropsychology, Maastricht University, Maastricht, the Netherlands

**Keywords:** Dementia, Prevention, Sociodemographic factors, Health literacy, Cross-sectional studies

## Abstract

**Background:**

Dementia is a leading cause of disability and mortality worldwide. While the disorder is widely recognized, public awareness of modifiable risk and potentially protective factors remains limited. This is despite evidence that a substantial proportion of cases could be prevented or delayed by modifying personal risk factors. To date, the influence of sociodemographic factors on knowledge about dementia prevention has not been sufficiently examined, particularly in Germany, leaving a critical gap for targeted public health strategies.

**Objectives:**

To assess awareness of the preventability of dementia and to evaluate knowledge of risk and protective factors in the German population, with particular focus on the influence of age, sex, and education.

**Design:**

Online, cross-sectional survey study.

**Setting:**

German population. A link to the survey was distributed nationwide via e-mail, flyers, and social media.

**Participants:**

Adults aged ≥18 years without diagnosed cognitive impairment. A total of 2610 individuals completed the survey, of whom 2515 (mean age 52.5 years, range 18–95, 69.8% female) were included in the analysis.

**Measurements:**

Awareness of dementia, risk factors, and preventability was assessed using two dichotomous and three Likert-scale items. Knowledge of 23 evidence-based risk and protective factors (plus sham items) was measured with Likert-scale items. Composite knowledge scores were derived from these items, including separate subscores for medical and lifestyle-related risk factors. Preferred information dissemination sources were assessed using a multiple-choice item. Analyses included descriptive statistics and regression models with age, sex, and education as predictors.

**Results:**

While almost all respondents (98.2%) affirmed knowing what dementia is, only 73% affirmed awareness of risk-modifying factors, with substantial subgroup differences. Nearly 38% did not agree that dementia can be prevented, including a higher proportion of those aged ≥75 years (52%). Lifestyle factors, such as physical, mental, and social activity and diet, were most frequently recognized (>75%), whereas medical and environmental risks (e.g., cardiovascular disease, kidney disease, air pollution) were consistently underrecognized (<50%). Overall, younger age, female sex, and higher education were predictors of significantly higher knowledge scores, with education showing the strongest effect. Preferred information sources also differed systematically; lower-educated participants and men were more likely to rely on general practitioners, while higher-educated groups preferred digital resources and specialized organizations.

**Conclusions:**

Compared with findings from previous German surveys, awareness of dementia preventability is higher in the present sample; however, knowledge about specific influencing factors—particularly medical—remains limited. As awareness, knowledge, and preferred information channels differ across age, sex, and education groups, educational efforts should be tailored accordingly.

## 1 Introduction

Dementia is a major global public health challenge, with prevalence and societal costs projected to rise sharply in the coming decades [1,2]. At the individual level, the disorder severely affects life expectancy and quality of life, and introduces significant caregiver burden [3,4]. Meanwhile, curative treatments remain unavailable [5]. In this context, the prevention of dementia is increasingly the focus of scientific interest. Recent estimates by the standing Lancet Commission on Dementia Prevention, Intervention and Care suggest that nearly half (45 %) of dementia cases could be prevented or at least significantly delayed by eliminating 14 risk factors [6]. In their model, dementia prevention is to be understood in a life-long perspective, characterized by different emphases in different stages of life, beginning in childhood. In early life, a high-quality education plays a particularly central role, as it substantially contributes to the development of cognitive reserve and has been shown to exert a significant influence on cognitive status in older age [7]. While education is the most relevant early-life factor, cognitively stimulating activity in adulthood is also considered part of this broader construct, with importance for reducing dementia risk [6,8,9]. In mid-adulthood, medical (i.e., hearing loss, elevated cholesterol, depression, traumatic brain injury, diabetes, and hypertension) and lifestyle-related risk factors (i.e., physical inactivity, smoking, obesity, and excessive alcohol use) become important. In later life, measures such as the maintenance of social activities, avoiding exposure to polluted air, and the early correction of vision loss are of particular importance [6]. Recent work extending the Lancet Commission framework further emphasizes that dementia risk factors are embedded in broader social and socioeconomic contexts. Structural conditions such as educational opportunities, economic resources, and access to healthcare shape both exposure to individual risk factors and their impact across the life course, thereby contributing not only to differences in prevalence but also to variations in effect sizes between population groups, for example between men and women [10].

The LIBRA (Lifestyle for Brain Health) Index and its updated version LIBRA2—a validated composite score that summarizes modifiable risk and protective factors for cognitive decline and dementia—integrates several of the Lancet Commission factors and adds additional modifiable components, namely unhealthy diet, chronic heart disease, chronic kidney disease, and sleep disturbances [11]. Beyond the variables included in the Lancet Commission model and LIBRA2 Index, accumulating evidence suggests an association between psychological stress and increased risk of cognitive impairment and dementia [12]. Finally, non-modifiable determinants, including age, sex, and parental history of dementia, are consistently recognized contributors [13,14].

Dementia-specific health literacy describes the ability to obtain, comprehend, and use information relevant to brain health and dementia prevention [15,16]. In this context, education and targeted knowledge dissemination represent key steps in enabling health-conscious lifestyles that can strengthen brain health and reduce dementia risk over the long term. Based on recent systematic reviews, the International Research Network on Dementia Prevention (IRNDP) has developed a consensus statement on priorities for future research. The network described the inadequate evidence on public knowledge about dementia prevention—particularly with respect to the more detailed investigation of differences between population subgroups—as a crucial deficit in dementia research. In addition, they describe a lack of knowledge transfer and prevention programs that could contribute to the promotion of (brain-)healthier lifestyles at both societal and individual levels [17].

Various international studies from recent years demonstrate the clear need for such measures. For example, a Dutch survey found that only 44 % of respondents considered it possible to reduce their own dementia risk [18], with a UK study reporting an even lower level of awareness at 36 % [19]. Knowledge about the influence of medical factors is particularly limited; a Norwegian study showed, for example, that diabetes and hearing loss were identified as risk factors by only a minority of respondents (26 % and 18 %, respectively) [20]. Sociodemographic variables appear to play an important role in shaping such knowledge, with higher educational attainment consistently associated with greater awareness of dementia prevention [[18], [19], [20], [21]], suggesting a dual role of education as a determinant of both dementia risk and dementia prevention knowledge. Women also tend to report higher levels of dementia-related knowledge [20,22,23], whereas findings regarding the influence of age have been less consistent [18,19,[21], [22], [23], [24], [25]] . A Danish awareness campaign carried out between 2021 and 2023 further emphasized that successful knowledge transfer requires messages adapted to specific subgroups, for example targeting age, sex, or educational level [26]. A review by Cations et al. identified considerable international variation in dementia-prevention knowledge, emphasizing the need for country-specific data [27].

For the German population, two studies to date have examined public knowledge about the preventability of dementia. In a study from 2012 among adults aged 18 years and older (*n* = 1 002), 55 % of respondents believed dementia prevention was not possible; in an open-ended question, fewer than half were able to name protective factors, such as memory training (47 %), mental activity (34 %), physical exercise (14 %), or social contact (13 %) [28]. A more recent study, from 2022 and restricted to individuals aged 60 years and older (*n* = 500), found that 68 % of participants considered dementia risk to be modifiable; in closed questions, physical and cognitive activities were each identified as protective by 87 %, and social isolation was reported as the most relevant risk factor (82 %) [23]. Since the 2012 study did not differentiate between age groups, it remains unclear whether these differences reflect an increase in knowledge over time or whether older adults might be generally better informed about dementia and possibilities for its prevention. Overall, this shows that the data regarding public knowledge about the preventability of dementia in Germany is currently inadequate, particularly with respect to differences across sociodemographic subgroups. This leaves an important gap for the development of targeted prevention and education strategies.

This study therefore aims to provide a detailed analysis of the level of knowledge regarding dementia-related risk factors in the German population, with a particular focus on the influence of age, sex, and education. In addition, it examines which information channels people prefer for receiving knowledge about dementia prevention. In this way, the study seeks to provide an important foundation for tailored communication strategies for dementia prevention. Such strategies could promote increased awareness of risk and protective factors for dementia across age and other population subgroups.

## 2 Methods

### 2.1 Study design and recruitment

An online survey was developed for a cross-sectional study of the German population. Recruitment took place between June 21 and December 15, 2024. A link and QR code to the survey were distributed through various channels: social networks (Instagram, WhatsApp, Facebook, nebenan.de), emails to associations throughout Germany (sports clubs, cultural associations, etc.), and on flyers in areas such as general practitioners’ offices or pedestrian zones.

Participants had to be at least 18 years old. A self-reported previous diagnosis of cognitive impairment was an exclusion criterion. Participation was voluntary, anonymous, and possible only once per participant via the web-based tool SoSci Survey. The survey addressed awareness of the modifiability of dementia risk and knowledge about specific influencing factors. Completion of the survey took approximately ten minutes. The study was registered in the German Clinical Trials Register (DRKS00033210). Ethical approval was obtained from the Ethics Committee of the Faculty of Medicine of the University of Cologne in September 2023.

### 2.2 Measurements

Questions on sociodemographic data included age, sex, and educational background. Participants were categorized into five age groups (18–29, 30–49, 50–64, 65–74, and 75 and older). Sex was defined as male, female, or diverse. However, due to the small number of participants (*n* = 15) in the “diverse” group, sex was analyzed as a dichotomous variable (male/female) to ensure sufficient statistical power and model stability. Educational background was assessed through questions on the highest school-leaving certificate and vocational qualification. Based on these, three groups were created: “high education” (completed or ongoing university studies), “medium education” (upper secondary school diploma but no ongoing or completed university studies), and “low education” (neither upper secondary school diploma nor university studies).

To assess awareness about dementia itself, two closed items (yes/no) asked whether participants knew what dementia means and whether they knew factors that influence risk. Participants’ awareness regarding the modifiability of dementia risk was then assessed using three statements: “There is nothing you can do to reduce your own dementia risk,” “Dementia is part of the normal aging process,” and “Dementia cannot be prevented.” Knowledge of risk and protective factors for dementia was assessed by asking: “To what extent do you believe that the following factors influence the risk of dementia?” For the three statements and for the questions on influencing factors, participants could choose from the response options “strongly agree,” “somewhat agree,” “somewhat disagree,” “strongly disagree,” and “I don’t know.”

A total of 23 influencing factors were included in the analysis. Of these, 14 were from the framework established by the Lancet Commission, namely education, hearing loss, elevated cholesterol, depression, traumatic brain injury, physical activity, diabetes, smoking, hypertension, obesity, excessive alcohol, frequent social contact, air pollution, and vision loss [6]. While the Lancet Commission combines school education and mental activity into a single factor, our study assessed them separately, given that mental activity represents a more modifiable aspect over a lifetime. In addition, four further factors, established in the LIBRA2 Index, were considered: healthy diet, chronic heart disease, chronic kidney disease, and sleep disturbances [11]. Psychological stress was also included, given evidence of its association with cognitive impairment and dementia risk [12]. Finally, three non-modifiable determinants were considered: sex, age, and parental history of dementia [13,14]. Additional “sham items” (i.e., factors with little or no scientific evidence, such as mobile phone radiation, aluminum, or excessive computer gaming) were included to detect or rule out a monotonic response tendency among participants.

Furthermore, a multiple-choice question was used to assess which information channels participants would prefer to use if they required information on dementia and dementia prevention. Response options included internet research, general practitioner, library, scientific publications, specialized organizations, medical specialist, self-help organizations, and “I don’t know”.

An English version of the complete questionnaire is provided in the Supplementary Materials.

### 2.3 Score construction

A multistage scoring system was developed to quantitatively assess knowledge about dementia prevention in the general population. This was initially based on the full set of 23 established risk factors derived from current international research, comprising both modifiable and non-modifiable factors (“overall score”). To allow for a more targeted analysis of prevention-relevant knowledge, an additional focused score was created that included only the 14 modifiable risk factors identified by the Lancet Commission [6] (“Lancet score”). For further differentiation, this 14-point score was divided into two thematic subscores: the “Lancet medical subscore”, comprising seven medical factors (hearing loss, elevated cholesterol, depression, traumatic brain injury, diabetes, hypertension, and vision loss) and the “Lancet lifestyle subscore”, comprising seven lifestyle factors (mental activity [in place of education], physical activity, smoking, obesity, alcohol use, social activity, and air pollution). This distinction between “medical” and “lifestyle” factors should not be interpreted as a strict conceptual or causal separation, but rather as a pragmatic approach to differentiate knowledge patterns while providing a comprehensible overview. For each correctly recognized (“strongly agree” or “somewhat agree”) factor, one point was awarded, allowing participants to achieve scores between 0 and 23 (“overall score”), 14 (“Lancet score”), or 7 for each of the two subscores.

### 2.4 Statistical analysis

All analyses were conducted using IBM SPSS Statistics 27. First, a detailed descriptive analysis of the sample characteristics and all collected variables was performed, reporting frequencies, ranges, means, and standard deviations, as appropriate.

For the three questions on awareness regarding the modifiability of dementia risk, the responses “strongly disagree” and “somewhat disagree” were combined and classified as correct (i.e., showing awareness of preventability), whereas “strongly agree,” “somewhat agree,” and “I don’t know” were classified as incorrect. Factors influencing the risk of dementia were classified as correctly recognized if the responses “strongly agree” or “somewhat agree” were given; the responses “somewhat disagree,” “strongly disagree,” and “I don’t know” were combined and classified as “not recognized.”

Differences in response rates between groups were examined using binary logistic regression analyses (for single items) and linear regression analyses (for mean scores) to determine the influence of age groups, sex, and educational level on knowledge about dementia and dementia prevention. *P*-values < 0.05 were considered statistically significant in two-sided tests. Statistical trends with *p*-values < 0.10 are also indicated in figures.

The question on preferred information channels was analyzed descriptively, presenting relative frequencies with additional consideration of the sociodemographic categories mentioned above.

## 3 Results

### 3.1 Sample characteristics

The total sample consisted of *n* = 2 610 participants. Seventy-seven persons were excluded due to a self-reported prior diagnosis of cognitive impairment, three due to missing sociodemographic data, and 15 who identified as diverse genders, as this group was too small to be analyzed. Thus, the analyzed sample consisted of *n* = 2 515 participants. Participants ranged in age from 18 to 95 years (*M* = 52.5; SD = 15.9). Of the total sample, 69.8 % were female (*n* = 1 756) and 30.2 % were male (*n* = 759). Regarding educational level, 55.7 % of respondents were classified as the high education group, 23.7 % as the medium education group, and 20.6 % as the low education group.

A more detailed characterization of the sample is provided in Table 1.

**Table 1:** Sample characteristics.

**Characteristic**	**mean (SD)**	**n**	**%**
**Age** (years)	52.5 (15.9)	2515	100
18–29		307	12.2
30–49		611	24.3
50–64		995	39.6
65–74		449	17.9
75 and older		153	6.1
**Sex**		2515	100
female		1756	69.8
male		759	30.2
**Educational level**		2515	100
low		518	20.6
medium		596	23.7
high		1401	55.7

### 3.2 General awareness of dementia and its preventability

Overall, 98.2 % of participants affirmed that they had an idea of what dementia is. In contrast, only 73 % responded “yes” when asked whether they knew of factors influencing dementia risk, with significant subgroup differences: Only 64.2 % of 18–29-year-olds affirmed such knowledge, whereas all older age groups exceeded 70 %, peaking at 76.1 % (65–74 years) and 77.6 % (≥ 75 years; both *p* < 0.001). Significant differences were also found by sex, with 76 % of women vs. 66 % of men responding affirmatively (*p* < 0.001). Higher education was associated with more affirmative responses (low 60.3 %, medium 70.8 %, high 78.6 %; *p* < 0.001).

Three statements examined awareness of risk modifiability, for which disagreement was considered a correct answer. The first statement (“There is nothing you can do to reduce your own dementia risk”) was answered correctly by 79.2 %, the second (“Dementia is part of the normal aging process”) by 70.9 %, and the third (“Dementia cannot be prevented”) by 63.4 %. Age effects were only significant for the third statement, with correct responses being lowest among participants aged 75 years and older (48.4 %; *p* = 0.009) compared with 63.7 %–65.7 % in the other groups. Women answered the first two statements correctly more often than men (80.6 % vs. 75.9 %, *p* = 0.007; 73.7 % vs. 64.5 %, *p* < 0.001), with no difference for the third (63.9 % vs. 61.9 %, *p* = 0.389). Higher education was consistently associated with more correct responses (first statement: high 83.8 %, medium 77.8 %, low 68.1 %, *p* < 0.001; second: 72.6 %, 69.0 %, 68.4 %, *p* = 0.04; third: 67.4 %, 62.3 %, 53.6 %, *p* < 0.001).

Detailed results for these analyses are provided in the Supplementary Material (Table S1).

### 3.3 Knowledge about dementia risk and protective factors

**Fig. 1:**
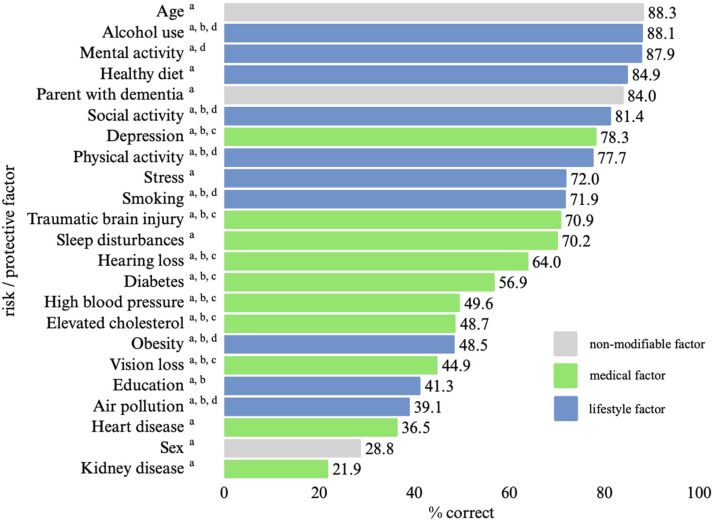
Percentage of participants correctly identifying each risk or protective factor, overall sample. ^a^ factor included in the overall score ^b^ factor included in the Lancet score ^c^ factor included in the Lancet medical subscore ^d^ factor included in the Lancet lifestyle subscore.

From the 23 risk factors assessed, those most frequently recognized as associated with dementia risk were age (88.3 %), alcohol use (88.1 %), mental activity (87.9 %), healthy diet (84.9 %), parental history of dementia (84.0 %), and social activity (81.4 %). The least frequently recognized factors were education (41.3 %), air pollution (39.1 %), heart disease (36.5 %), sex (28.8 %), and kidney disease (21.9 %) (Fig. 1).

Ten of the 23 factors were most frequently recognized by the youngest age group (18–29 years): age (*p* = 0.002), alcohol use (*p* = 0.003), mental activity (*p* = 0.002), parental history of dementia (*p* < 0.001), stress (*p* < 0.001), traumatic brain injury (*p* < 0.001), sleep disturbances (*p* < 0.001), heart disease (*p* < 0.001), sex (*p* < 0.001), and kidney disease (*p* = 0.001). Participants in middle age most often recognized depression (*p* = 0.003), diabetes (*p* = 0.01), and school education (*p* < 0.001). Older participants more often identified social activity (*p* = 0.021), physical activity (*p* = 0.015), hearing loss (*p* < 0.001), and vision loss (*p* = 0.002) as risk or protective factors.

**Fig. 2:**
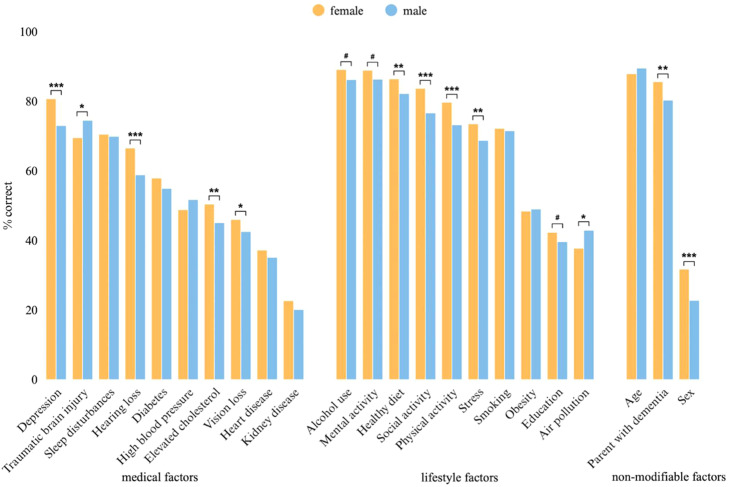
Percentage of participants correctly identifying each risk or protective factor, by sex. * *p* < 0.05; ** *p* < 0.01; *** *p* < 0.001. # *p* < 0.1.

Women recognized ten of the 23 factors significantly more often than men: healthy diet (86.3 % vs. 82.1 %, *p* = 0.006), parental history of dementia (85.5 % vs. 80.2 %, *p* = 0.003), social activity (83.6 % vs. 76.5 %, *p* < 0.001), depression (80.6 % vs. 72.9 %, *p* < 0.001), physical activity (79.6 % vs. 73.1 %, *p* < 0.001), stress (73.4 % vs. 68.6 %, *p* = 0.006), hearing loss (66.4 % vs. 58.7 %, *p* < 0.001), elevated cholesterol (50.3 % vs. 45.0 %, *p* = 0.008), vision loss (45.9 % vs. 42.4 %, *p* = 0.027), and sex (31.6 % vs. 22.6 %, *p* < 0.001). Men more frequently recognized traumatic brain injury (74.4 % vs. 69.4 %, *p* = 0.012), and air pollution (42.8 % vs. 37.6 %, *p* = 0.017) (Fig. 2).

**Fig. 3:**
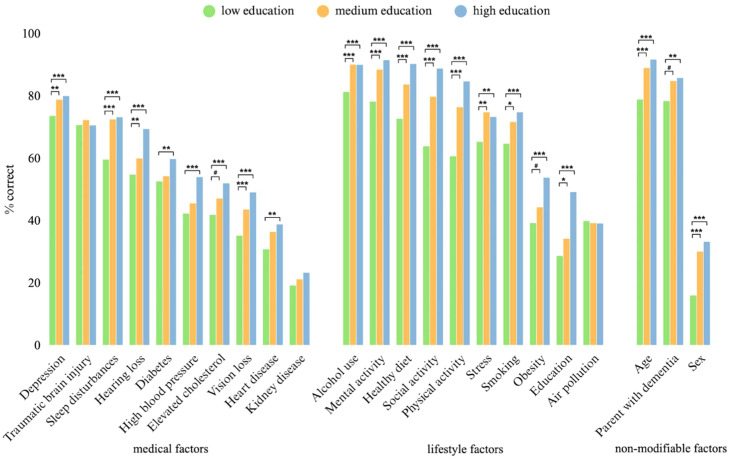
Percentage of participants correctly identifying each risk or protective factor, by educational level. * *p* < 0.05; ** *p* < 0.01; *** *p* < 0.001. # *p* < 0.1.

Participants in the lowest education group recognized all factors—except for traumatic brain injury, air pollution, and kidney disease—significantly less often than those in the highest. The largest differences were observed for healthy diet (90.2 % vs. 72.6 %, *p* < 0.001), social activity (88.7 % vs. 63.8 %, *p* < 0.001), physical activity (84.6 % vs. 60.6 %, *p* < 0.001), and school education (49.1 % vs. 28.6 %, *p* < 0.001) (Fig. 3).

For the sham items with unproven links to dementia risk, 12.3 % of participants incorrectly selected mobile phone radiation, 22.2 % selected aluminum, and 26.6 % selected excessive computer gaming as factors influencing dementia risk.

Detailed results for these analyses are provided in the Supplementary Material (Table S2).

### 3.4 Mean knowledge scores

Across all participants, the mean overall score was 14.34 out of 23 (SD 4.82). The mean Lancet score was 8.60 out of 14 (SD 3.41), with 4.13 out of 7 (SD 2.04) for the Lancet medical subscore and 4.94 out of 7 (SD 1.68) for the Lancet lifestyle subscore.

Knowledge scores differed by age. Younger adults (18–29 years) achieved the highest mean overall score (*M* = 14.93, SD 4.64), but the lowest on the Lancet medical subscore (*M* = 3.92, SD 1.97). Meanwhile, mid- and older-age groups scored higher in the latter domain (*M* ≥ 4.18). For lifestyle factors, there was a tendency toward higher values among younger participants (*M* = 5.07 vs. 4.78 among those aged 60–74, SD 1.73 and 1.62, respectively), although this difference fell narrowly outside of statistical significance (*p* = 0.056). Significant sex and education effects were observed across all scores. Women had consistently higher mean values than men (e.g., 14.53 vs. 13.90 in the overall score, SD 4.79 and 4.87, *p* < 0.001). A pronounced stepwise increase was also evident with higher educational attainment (overall score: low education 12.42, medium 14.16, high 15.12; SD 5.27, 4.60, and 4.53, respectively; *p* < 0.001).

**Fig. 4:**
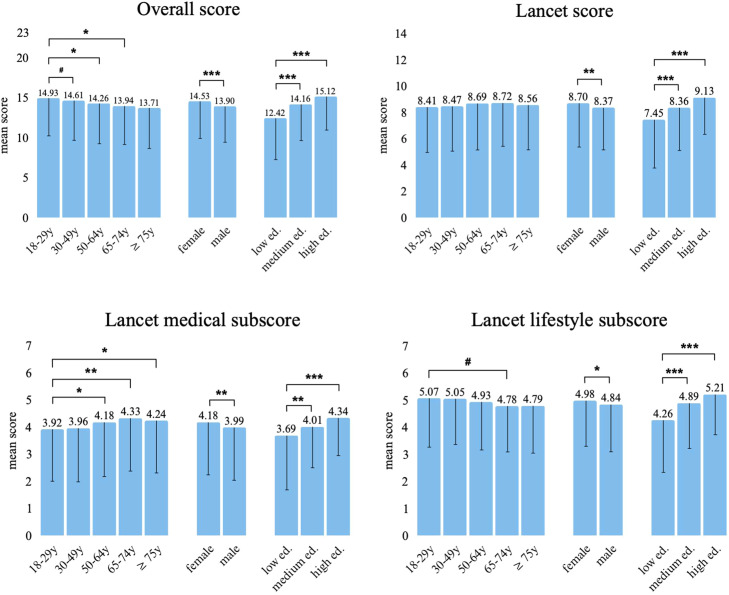
Mean knowledge scores by age group, sex, and educational level. Notes: Bars represent mean scores achieved by participants in each subgroup. Participants received one point for each correctly recognized dementia risk or protective factor. The overall score (0–23; overall mean = 14.34) includes all 23 assessed evidence-based factors. The Lancet score (0–14; overall mean = 8.60) includes only the 14 modifiable risk factors identified by the Lancet Commission, subdivided into the Lancet medical (0–7; overall mean = 4.13) and Lancet lifestyle (0–7; overall mean = 4.94) subscores. * *p* < 0.05; ** *p* < 0.01; *** *p* < 0.001. # *p* < 0.1.

All mean scores are presented in Fig. 4, with statistical details available in the Supplementary Material (Table S3).

### 3.5 Preferred sources of information on dementia prevention

In a multiple-choice question, participants were asked which sources they would use if they needed information on dementia prevention. Internet research was by far the most frequently preferred source (83.8 %), followed by specialized organizations such as the German Alzheimer Society (58.7 %), medical specialists (56.4 %), and general practitioners (43.7 %). Scientific publications (36.8 %), self-help organizations (27.9 %), and libraries (14.4 %) were selected less frequently; 1.4 % responded “I don’t know.”

Clear age differences were observed. Internet use was most frequently preferred among 18–29-year-olds (90.6 %) and declined steadily with age, reaching 57.5 % among participants aged 75 and older. In contrast, consultation with medical specialists (60.1 % vs. 47.6 %) and general practitioners (45.8 % vs. 38.4 %) was preferred in the oldest age group compared with the youngest. Specialized organizations and self-help groups were most often selected by participants aged 50–64 (63.2 % and 30.6 %, respectively) and 65–74 (63.0 % and 29.9 %, respectively), and remained comparatively high even in the oldest group (54.9 % and 24.8 %, respectively). These channels were least often chosen by the youngest group (45.7 % and 19.0 %, respectively).

Internet research was preferred almost equally by women (84.0 %) and men (83.3 %), as was consultation with medical specialists (56.1 % and 56.9 %, respectively). Men more often opted for general practitioners (49.3 % vs. 41.3 %), whereas women more frequently chose specialized organizations (62.4 % vs. 50.1 %) and self-help organizations (30.9 % vs. 20.9 %).

Educational differences were pronounced: Internet research was preferred by 87.4 % of participants with high education compared with 71.8 % with low education. Similar patterns were observed for scientific publications (42.1 % vs. 26.4 %) and specialized organizations (61.4 % vs. 53.2 %). Conversely, general practitioners were more frequently opted for by participants with lower education (51.4 % vs. 40.1 %).

## 4 Discussion

### 4.1 Key findings

Our survey investigated awareness of possibilities for dementia prevention and knowledge about risk-influencing factors in the German population, focusing on the influence of age, sex, and education. The results indicate that both awareness of dementia preventability and knowledge about specific risk factors—particularly medical—are limited and unevenly distributed across sociodemographic groups. Preferences for information channels likewise varied substantially between these groups.

Dementia was confirmed as widely recognized in Germany, with almost all participants stating that they had an idea of what the condition means. This is consistent with studies reporting that participants largely reliably recognize dementia symptoms in case vignettes [29,30]. Although nearly 30 % of respondents in our sample believed dementia is a normal part of the aging process, this is lower than in previous international reports [27], suggesting comparatively higher awareness that dementia is not an inevitable consequence of aging. In addition, almost 80 % of participants disagreed with the statement that “there is nothing you can do to reduce your own dementia risk,” indicating a higher level of openness to the idea that individuals influence their risk, as earlier international studies reported values between 36 % and 70 % [19– 22].

Nonetheless, these more positive perceptions were not equally distributed across the population. Individuals with lower educational attainment were significantly less likely to reject the statement that nothing can be done to reduce dementia risk, showing social gradients in beliefs about dementia preventability. More than one-third of respondents did not agree that dementia can be prevented, underscoring that a fatalistic view of dementia development remains widespread in Germany. This was particularly evident among adults aged 75 years and older, more than half of whom were unaware of the possibility of dementia prevention. This pattern mirrors findings from the Netherlands and Denmark, where older adults stated that dementia cannot be prevented more often than younger people [18,26]. Such a belief may lead to less interest in opportunities for dementia prevention—as people are unaware of their existence and relevance—less attention to early symptoms, and lower use of professional services for diagnosis and treatment [31,32]. Conversely, knowledge of the modifiability of one’s own dementia risk can contribute to healthier lifestyle decisions, and prevention should be understood as a lifelong process that remains highly relevant in later life [6].

However, to live a “brain-healthy” lifestyle and achieve the best possible dementia prevention, it is necessary to know which factors actually contribute; here, substantial gaps in knowledge become apparent. Knowledge of medical risk factors lagged considerably behind that of more intuitive factors and those more frequently highlighted in the media, such as age, mental activity, and healthy diet. Accordingly, the mean value for the Lancet medical subscore was lower than that of the lifestyle equivalent. This finding aligns with studies from Iceland, New Zealand, Norway, and another from Germany, which also found that “mental activity,” “physical activity,” “social activity,” and “healthy diet” were consistently most frequently recognized [20,22,23,25]. Risk factors such as chronic heart and kidney disease were rarely recognized in any previous surveys, with values around 10 %–30 % [20,22,23] Especially striking—in our survey and others—are the low recognition rates for metabolic syndrome factors (elevated cholesterol, diabetes, hypertension, and obesity), which are highly prevalent in the Western world (19 % of German adults are obese [33] and 29.3 % have hypertension [34]). Since these factors are now part of routine medical care in many countries, including Germany, they represent major opportunities for informing people about dementia risk reduction.

Across international studies to date, no consistent directional effect of age has been observed on knowledge about dementia prevention [20,22,25,26]. Interestingly, participants aged 18–29 rated their own knowledge of risk-relevant factors lowest in our survey, but achieved the highest results in the overall score of all 23 evaluated factors. This may indicate that younger individuals possess a higher level of knowledge regarding “general health behavior,” but do not apply this knowledge specifically to dementia, which is often perceived as a disease of old age. Younger individuals may be less motivated to engage in behavioral changes that reduce dementia risk, as the issue may appear too distant or irrelevant at their stage of life [35,36]. Yet this stands in contrast to current approaches that emphasize the importance of early-life prevention, therefore often framed as “promoting brain health” [37]. More detailed analysis shows that our younger participants had greater knowledge of certain factors—often conveyed through the media or school—such as alcohol use, stress, or sleep disturbances, while displaying lesser knowledge of medical determinants. Older respondents, in contrast, more often correctly recognized medical risk factors, perhaps attributable to personal experiences of disease and medical consultations or counseling. In middle adulthood, peak values were observed for factors such as diabetes and depression, conditions more frequently diagnosed or discussed during this stage of life [38,39]. As outlined earlier, previous German data [23,28] did not allow conclusions regarding potential age-related differences in dementia-prevention knowledge. Our findings now provide evidence that knowledge about dementia prevention is not uniform across the population, but varies by topic and reflects different age-related priorities.

Striking differences in knowledge levels between the sexes were observed. Women achieved higher mean values across all scores and were more likely to correctly recognize lifestyle-related and psychosocial determinants, such as diet, social activity, depression, and stress. This is consistent with a broad body of international evidence attributing to women a greater interest in preventive health behaviors and a stronger orientation toward health-promoting lifestyles [40,41]. Men, in contrast, recognized only a few factors more often, such as traumatic brain injury and air pollution; these determinants are frequently associated with highly visible individual events or acute hazard scenarios. This may indicate that men respond more strongly to specific health risks that are personally experienced or publicly discussed, and may tend to attribute health risks to external circumstances rather than personal factors.

The effects of education were particularly pronounced in the results, with more highly educated participants showing higher general awareness of dementia preventability and specific risk factors, reflected in higher values in all four knowledge scores. These findings align with extensive evidence from numerous international studies [18,20– 22,26]. Differences between educational groups were particularly pronounced for lifestyle factors. Knowledge of medical risk factors may be more likely to be conveyed during medical consultations and therefore less dependent on formal education, whereas lifestyle-related knowledge requires self-directed information seeking. This finding is relevant from two perspectives: first, higher education is typically associated with greater health literacy, better access to evidence-based information, and a more reflective approach to health risks [42]. Second, it points to a double risk for people with lower education levels: lower knowledge about a brain-healthy lifestyle coincides with the well-documented association between a lower educational level and increased dementia risk [6]. As educational attainment is positively associated with socioeconomic background [43], this pattern highlights that both the development of risk factors and prevention-related knowledge and behavior need to be understood within this broader socioeconomic context.

The analysis of preferred information channels also provides important insights for targeted knowledge dissemination. Although internet research was the most frequently selected source across all groups, striking differences were observed: younger respondents clearly preferred digital sources of information, whereas older individuals were more likely to rely on personal counseling from specialists and general practitioners. This finding is consistent with current evidence demonstrating age-dependent differences in health information-seeking behavior [44]. Men and individuals with lower education levels—groups with poorer knowledge about dementia prevention—also more often chose general practitioners as their preferred source of information. Women and participants with higher education were more likely to choose scientific publications, specialized organizations, and self-help groups. Critically, this indicates that people with a lower interest in health-promoting behavior are less likely to proactively and independently seek health-related information, even though they would benefit from it the most [45]. These patterns underscore that prevention messages must be tailored to the target groups, not only in terms of content but also in the choice of communication channels, taking into account the social and structural contexts that influence how prevention-related information is encountered and used. Primary care structures may play a key role by integrating outreach approaches into routine physician visits to reach population groups that are currently underserved with prevention-related information.

### 4.2 Strengths and limitations

The study has several methodological strengths. As one of the largest surveys of its kind [27], with more than 2 500 participants, it allowed for a differentiated analysis by age, sex, and education. Notably, the sample included adults from age 18, a younger range not covered in several previous studies. Drawing on internationally recognized sources, such as the Lancet Commission and LIBRA2 Index, ensured a high degree of comparability with existing studies. The inclusion and analysis of a large number of established risk factors enabled a comprehensive examination of the population’s knowledge of dementia prevention. The development of novel composite scores allowed for a clearer and more accessible representation of the results to indicate “dementia-specific health literacy”. Subdividing modifiable factors into medical and lifestyle subscores enabled further differentiation of knowledge patterns. This distinction should be understood as a pragmatic rather than a strictly conceptual separation, as many of the factors included are shaped by overlapping behavioral, social, and environmental influences across the life course. For example, factors primarily labelled as “medical” (e.g., hypertension) may be influenced in their onset, progression, and management by lifestyle-related behaviours such as diet, physical activity, or stress. Taken together, the resulting scores offer a comprehensible overview of existing knowledge gaps, reducing complexity and potential measurement inaccuracies by condensing individual responses into structured, comparable measures. This study therefore provides, for the first time in Germany, an empirical foundation for evidence-based targeting of the content and delivery of educational efforts, tailored to age-, sex-, and education-specific groups.

Limitations of this study include an overrepresentation of women and individuals with a higher level of education, likely due to the voluntary online survey format [46,47], as well as a potential participation bias, as individuals with a general interest in dementia prevention may have been more likely to take part. Consequently, the results cannot be generalized without restriction to the entire population. In addition, all measures were self-reported, and the binary scoring approach, in which “somewhat” and “strongly” (dis)agree responses (depending on item direction) were equally grouped as correct, may slightly overestimate knowledge levels. Moreover, classifying “I don’t know” responses as “not recognized” collapses uncertainty and disagreement, so that observed group differences may partly reflect variation in response confidence or perceived relevance rather than knowledge alone. Given the number of item-level analyses and the absence of adjustment for multiple comparisons, there is also a potential for inflated Type I error. Together, these aspects indicate that subgroup differences are best recognized as illustrative patterns, whereas absolute knowledge levels should be interpreted with caution. Participants with diverse gender identities could not be analyzed due to the small sample size. As previous research has shown an increased risk of dementia among transgender and non-binary individuals [48], future research should address this data gap.

### 4.3 Conclusion and implications for future interventions

Dementia is widely recognized in the German general population, and a substantial proportion of respondents in our survey acknowledge the possibility of influencing their individual risk through a health-conscious lifestyle and preventive medical measures. However, this awareness remains limited overall and is unevenly distributed across sociodemographic groups. The present findings indicate substantial knowledge gaps regarding risk and protective factors, particularly medical-related factors. People with higher educational levels consistently showed the best dementia-related knowledge. In addition, women and younger participants generally showed higher levels of knowledge, but considerable, topic-specific variation exists. In line with these differences, preferred sources for information on dementia prevention also varied according to age, sex, and educational level. To the best of the authors’ knowledge, there are currently no nationwide dementia prevention campaigns in Germany. This study therefore provides an empirical basis for future initiatives by offering practical guidance on how messages and interventions can be tailored to different population groups based on identified knowledge gaps.

In this context, an age-differentiated approach, both in terms of content and delivery, is necessary: Dementia prevention must take place across all life stages, starting in childhood, where equitable access to high-quality education must be ensured. Younger groups can be reached more effectively via social media and digital formats, and school-based outreach for children and adolescents. Meanwhile, for older adults, risk communication during medical consultation and personal counseling is essential. Furthermore, specialized organizations should provide an easily accessible and appealing online presence, and efforts should be made to raise awareness of their existence so they can serve as relevant and trustworthy sources of information.

Sex-specific patterns in both dementia risk factors and knowledge levels also call for differentiated strategies. Women are more frequently affected by factors such as lower educational attainment, depression, and insufficient physical activity [10], while diabetes [38], higher alcohol consumption [49], and reduced social activity [50] are more prevalent in men [10]. In men, these factors, combined with a general tendency toward less health-promoting behavior—also reflected in lower knowledge about dementia prevention—underline the need to specifically raise awareness of the importance of personal responsibility and engagement in dementia prevention through health-promoting behaviors.

The pronounced effects of education levels on dementia-related knowledge underscore that prevention strategies and educational efforts must reach all groups equally, with particular attention on individuals with lower formal education. To achieve this, both the language and the format of educational materials should be adapted accordingly, for example through clear and simple wording, visual support elements, and outreach services that do not require individual initiative. Examples of such public health initiatives exist from the Netherlands [51], Belgium [52], and Denmark [26]. For instance, the Netherlands Dementia Prevention Initiative has used extensive co-creation for raising knowledge and awareness in people with low socio-economic status or a migrant background, including adaptation of the app 'MyBraincoach' (MijnBreincoach) to disseminate knowledge and advice [53]. Furthermore, general practitioners and medical specialists should play a central and active role in targeted health communication, especially to groups that might otherwise remain underserved. However, many health professionals themselves require additional training on dementia prevention that both strengthens their knowledge and provides practical tools, such as conversation starters, to support the communication of dementia risk reduction in routine clinical practice [54]. Future research should examine whether these evidence-informed recommendations prove to be effective when implemented in targeted intervention strategies.

Overall, the limited public knowledge identified in this study indicates a risk that major opportunities for dementia prevention are being missed, particularly in vulnerable groups. Interventions tailored to age-, sex-, and education-specific needs in terms of content and choice of communication channels are therefore urgently needed to enhance dementia literacy and promote health-conscious behavior across the population. Strengthening the understanding that dementia risk can be substantially reduced through lifestyle choices and adequate management of medical conditions will be critical for effective prevention at the societal level.

## Funding

This study did not receive any third-party funding. All costs incurred were covered by the budget resources of the Medical Psychology Department, Medical Faculty and University Hospital Cologne.

## Declaration of generative AI and AI-assisted technologies in the writing process

No generative AI was used for writing purpose.

## CRediT authorship contribution statement

**Pauline Albus:** Writing – review & editing, Writing – original draft, Visualization, Software, Project administration, Methodology, Investigation, Formal analysis, Data curation, Conceptualization. **Ann-Kristin Folkerts:** Writing – review & editing, Visualization, Supervision, Methodology, Investigation, Conceptualization. **Josef Kessler:** Writing – review & editing, Validation, Methodology. **Sebastian Köhler:** Writing – review & editing, Validation, Methodology. **Elke Kalbe:** Writing – review & editing, Visualization, Supervision, Software, Resources, Project administration, Methodology, Investigation, Formal analysis, Conceptualization.

## Declaration of competing interest

The authors declare the following financial interests/personal relationships which may be considered as potential competing interests: Elke Kalbe declares that she is co-author of the cognitive intervention series "NEUROvitalis" (ProLog, Cologne, Germany), but receives no corresponding honoraria. Ann-Kristin Folkerts declares that she is co-author of the cognitive intervention series "NEUROvitalis" (ProLog, Cologne, Germany), but receives no corresponding honoraria. Pauline Albus, Josef Kessler and Sebastian Köhler declare that they have no known competing financial interests or personal relationships that could have appeared to influence the work reported in this paper.

## Appendix Supplementary materials

Image, application 1
