# Supplementary material for: Sociodemographic differences in dementia prevention knowledge in Germany: Implications for targeted health communication
Source: J Prev Alzheimers Dis. 2026 Feb 28;13(5):100517. doi: 10.1016/j.tjpad.2026.100517 (PMC12966667; doi:10.1016/j.tjpad.2026.100517)
Supplement: Supplementary file 1 [file mmc1.docx]

**Supplementary materials**

**Contents:**

**Questionnaire:** Dementia: What is it? What do I know about it? 02

**Table S1:** General awareness of dementia and its preventability 08

**Table S2:** Knowledge about dementia risk and protective factors 10

**Table S3:** Mean knowledge scores 17

**Questionnaire - Dementia: What is it? What do I know about it?**

**PART I: Personal information**

What is your gender?

□ Female

□ Male

□ Diverse

How old are you?

I am ________ years old.

Indicate your country of origin.

□ Germany

□ Turkey

□ Poland

□ Syria

□ Romania

□ Italy

□ Russia

□ I am originally from another country: ____________________

Only if you are originally from another country: how many years have you been living in Germany?

For _______ years

In which federal state do you live?

______________________________

How large is the town you live in?

□ less than 5,000 inhabitants

□ 5,000 to 20,000 inhabitants

□ 20,000 to 100,000 inhabitants

□ more than 100,000 inhabitants

What is your marital status?

□ Single

□ In a relationship

□ Married/civil union

□ Divorced/separated

□ Widowed

Who do you currently live with?

□ Alone

□ With one other person

□ With other people (more than two)

□ Retirement home

Indicate your highest level of education.

□ No school-leaving certificate

□ Elementary school certificate

□ Elementary school certificate

□ Intermediate secondary school certificate

□ Entrance qualification for universities of applied sciences

□ General higher education entrance qualification

□ Other qualification, please specify: _______

What is the highest vocational qualification you have obtained?

□ No vocational qualification

□ Completed vocational training

□ Completed master craftsman or technician training

□ Bachelor’s degree

□ Master’s, Magister, or Diploma degree

□ Doctorate / Habilitation

□ Other vocational qualification, please specify: _______

How long did your vocational training last in total?

_________ years

Please indicate your current employment status (multiple answers possible):

□ Unskilled worker

□ Skilled worker

□ Employee

□ Self-employed

□ On parental leave

□ Homemaker

□ Not employed / Economically inactive

□ Retired

□ Other: ___________________________

Which job have you done for the longest period of time?

__________________________________________________________________________

If you indicated that you are retired, please indicate the type of pension you receive.

□ Old-age pension

□ Early retirement pension

□ Disability pension

How many years have you been retired?

For __________ years.

Do you suffer from hearing loss or impairment?

□ Yes

□ No

□ I don't know

Do you wear a hearing aid?

□ Yes

□ No

**PART II**

**With the following questions, we want to find out what you know about dementia.**

**We would also like to understand how much you think lifestyle and/or certain health problems can affect the risk of developing dementia.**

Do you have an idea of what dementia means?

□ Yes

□ No

When you think about dementia, what are the key points that come to mind?

__________________________________________________________________________

Here is a definition of dementia:

Dementia is a condition characterized by a progressive and persistent decline in cognitive functions, such as memory, attention, and the ability to express oneself. Mood can also be affected. Those affected gradually lose their independence and need increasing support from others. The most common form of dementia is Alzheimer's disease, but dementia can also occur in other neurological diseases, especially with advancing age.

How much do you agree with the following statements?

*“There is nothing you can do to reduce your own dementia risk.”*

□ Strongly disagree

□ Somewhat disagree

□ Somewhat agree

□ Strongly agree

□ I don't know

*“Dementia is part of the normal aging process.”*

□ Strongly disagree

□ Somewhat disagree

□ Somewhat agree

□ Strongly agree

□ I don't know

*“Dementia cannot be prevented.”*

□ Strongly disagree

□ Somewhat disagree

□ Somewhat agree

□ Strongly agree

□ I don't know

Do you know factors that increase or reduce the risk of dementia?

□ Yes

□ No

If yes, can you give some examples of factors that increase or reduce the risk of dementia?

_______________________________________________________________________________________

To what extent do you believe that the following factors influence the risk of dementia?

(Strongly disagree Somewhat disagree Somewhat agree Strongly agree Don't know)

High blood pressure

Parent with dementia

Use of painkillers

Being married

Alcohol use

Physical activity

Mobile phone radiation

Working in a noisy environment

Depression

Age

Genetic factors

Obesity

Poor personal care

Mental activity

Education

Kidney disease

Having children

Aluminum

Elevated cholesterol

Social activities

Healthy diet

Air pollution

Head injuries

Vitamin A deficiency

Cultural interest

Drug addiction/medication dependency

Sex

Heart disease

Smoking

Repetitive heading in football (soccer)

Diabetes

Traumatic brain injury

Loneliness

Hearing loss

Vision loss

Untreated tooth decay

Excessive computer gaming

Boxing

Stress

Religiousness/spirituality

Brain inflammation

Sleep disturbances

*Note: A total of 42 potential influencing factors were assessed. From these, only a selection was included in the analysis: 23 factors with the strongest empirical support, and 3 factors with little to no supporting evidence, which were included as clear sham items.*

Does anyone in your family or social circle have or have had dementia?

□ Yes

□ No

□ I don't know

If you wanted to find out more about dementia and how to prevent it, which sources of information would you use? You can select more than one option.

□ Internet research

□ General practitioner

□ Library

□ Scientific publications

□ Specialized organizations (e.g., Alzheimer's Association...)

□ Medical specialist

□ Self-help group

□ Other: _____________________________________________________________

□ I don't know

If someone is worried about developing dementia, who would they contact first for professional help?

□ I would contact: _____________

□ I don't know

**PART III: Personal information**

Do you feel that your memory or other cognitive functions are deteriorating?

□ Yes

□ No

□ I don't know

If yes, is this a cause for concern for you?

□ Yes

□ No

□ I don't know

Have you been diagnosed with cognitive impairment?

□ Yes

□ No

□ I don't know

If yes, how many years ago were you diagnosed with cognitive impairment?

□ _________ years ago.

□ I don't know

Are you doing activities that stimulate your cognitive abilities?

______________________________________________________________________________________________________________________________________________________________________________

**Table S1: General awareness of dementia and its preventability**

| Item | Subgroup | % "Yes" | % "No" |  | OR for "Yes" | 95% CI | *p* |
| --- | --- | --- | --- | --- | --- | --- | --- |
| **"Do you have an idea of what dementia means?"** | total | 98.2 | 1.8 |  |  |  |  |
|  | age 18-29 | 98.0 | 2.0 |  |  |  |  |
| dichotomous (yes / no) | age 30-49 | 98.2 | 1.8 |  |  |  |  |
|  | age 50-64 | 98.5 | 1.5 |  |  |  |  |
|  | age 65-74 | 98.0 | 2.0 |  |  |  |  |
|  | age ≥ 75 | 96.7 | 3.3 |  |  |  |  |
|  | female | 99.0 | 1.0 |  |  |  |  |
|  | male | 96.3 | 3.7 |  |  |  |  |
|  | low education | 97.3 | 2.7 |  |  |  |  |
|  | medium education | 97.8 | 2.2 |  |  |  |  |
|  | high education | 98.6 | 1.4 |  |  |  |  |
|  |  |  |  |  |  |  |  |
| **"Do you know factors that influence dementia risk?"** | total | 73.0 | 27.0 |  |  |  |  |
|  | age 18-29 | 64.2 | 35.8 |  |  |  |  |
| dichotomous (yes / no) | age 30-49 | 73.7 | 26.3 |  | 1.407 | 1.036, 1.910 | 0.029 |
|  | age 50-64 | 73.1 | 26.9 |  | 1.523 | 1.149, 2.020 | 0.003 |
|  | age 65-74 | 76.1 | 23.9 |  | 1.922 | 1.380, 2.679 | < 0.001 |
|  | age ≥ 75 | 77.6 | 22.4 |  | 2.505 | 1.577, 3.981 | < 0.001 |
|  | female | 76.0 | 24.0 |  |  |  |  |
|  | male | 66.0 | 34.0 |  | 0.577 | 0.476, 0.700 | < 0.001 |
|  | low education | 60.3 | 39.7 |  |  |  |  |
|  | medium education | 70.8 | 29.2 |  | 1.760 | 1.361, 2.277 | < 0.001 |
|  | high education | 78.6 | 21.4 |  | 2.647 | 2.115, 3.314 | < 0.001 |

| Item | Subgroup | % correct  ("somewhat disagree" + "strongly disagree") | "somewhat agree" + "strongly agree" | % "I don't know" | OR for  correct answer | 95% CI | *p* |
| --- | --- | --- | --- | --- | --- | --- | --- |
| **"There is nothing you can do to reduce your own dementia risk."** | total | 79.2 | 15.0 | 5.8 |  |  |  |
|  | age 18-29 | 78.8 | 15.3 | 5.9 |  |  |  |
| Likert (4-point + I don't know) | age 30-49 | 80.2 | 14.4 | 5.4 | 0.996 | 0.705, 1.406 | 0.981 |
|  | age 50-64 | 80.2 | 13.8 | 6.0 | 1,099 | 0.797, 1.515 | 0.565 |
|  | age 65-74 | 79.1 | 15.1 | 5.8 | 1,058 | 0.735, 1.523 | 0.761 |
|  | age ≥ 75 | 69.3 | 24.8 | 5.9 | 0.724 | 0.462, 1.135 | 0.159 |
|  | female | 80.6 | 14.7 | 4.7 |  |  |  |
|  | male | 75.9 | 15.7 | 8.4 | 0.751 | 0.609, 0.925 | 0.007 |
|  | low education | 68.1 | 19.2 | 12.7 |  |  |  |
|  | medium education | 77.8 | 17.3 | 4.9 | 1.622 | 1.236, 2.129 | < 0.001 |
|  | high education | 83.8 | 12.6 | 3.6 | 2.402 | 1.895, 3.045 | < 0.001 |
|  |  |  |  |  |  |  |  |
| **“Dementia is part of the normal aging process.”** | total | 70.9 | 26.4 | 2.7 |  |  |  |
|  | age 18-29 | 67.8 | 29.3 | 2.9 |  |  |  |
| Likert (4-point + I don't know) | age 30-49 | 71.7 | 26.0 | 2.3 | 1.115 | 0.825, 1.506 | 0.480 |
|  | age 50-64 | 72.3 | 25.7 | 2.0 | 1.192 | 0.901, 1.577 | 0.218 |
|  | age 65-74 | 68.6 | 26.5 | 4.9 | 1.037 | 0.755, 1.423 | 0.824 |
|  | age ≥ 75 | 71.9 | 26.8 | 1.3 | 1.296 | 0.842, 1.997 | 0.239 |
|  | female | 73.7 | 24.0 | 2.3 |  |  |  |
|  | male | 64.5 | 32.1 | 3.4 | 0.652 | 0.542, 0.784 | < 0.001 |
|  | low education | 68.4 | 25.4 | 6.2 |  |  |  |
|  | medium education | 69.0 | 29.0 | 2.0 | 1.033 | 0.798, 1.337 | 0.806 |
|  | high education | 72.6 | 25.8 | 1.6 | 1.263 | 1.011, 1.579 | 0.040 |
|  |  |  |  |  |  |  |  |
| **"Dementia cannot be prevented.”** | total | 63.4 | 30.4 | 6.2 |  |  |  |
|  | age 18-29 | 63.8 | 32.3 | 3.9 |  |  |  |
| Likert (4-point + I don't know) | age 30-49 | 65.7 | 28.4 | 5.9 | 1.041 | 0.779, 1.391 | 0.786 |
|  | age 50-64 | 63.7 | 29.8 | 6.5 | 1.011 | 0.773, 1.323 | 0.936 |
|  | age 65-74 | 64.1 | 29.2 | 6.7 | 1.042 | 0.767, 1.416 | 0.793 |
|  | age ≥ 75 | 48.4 | 42.4 | 9.2 | 0.590 | 0.396, 0.878 | 0.009 |
|  | female | 63.9 | 30.6 | 5.5 |  |  |  |
|  | male | 61.9 | 30.2 | 7.9 | 0.924 | 0.773, 1.106 | 0.389 |
|  | low education | 53.6 | 33.1 | 13.3 |  |  |  |
|  | medium education | 62.3 | 32.5 | 5.2 | 1.389 | 1.090, 1.771 | 0.008 |
|  | high education | 67.4 | 28.5 | 4.1 | 1.721 | 1.396, 2.120 | < 0.001 |

**Table S2: Knowledge about dementia risk and protective factors**

| Risk / protective factor | Subgroup | "somewhat agree" + "strongly agree" | "somewhat disagree" + "strongly disagree" | % "I don't know" | OR for  "somewhat agree" + "strongly agree" | 95% CI | *p* |
| --- | --- | --- | --- | --- | --- | --- | --- |
| **Age** | total | 88.3 | 9.1 | 2.6 |  |  |  |
|  | age 18-29 | 94.2 | 5.1 | 0.7 |  |  |  |
| Overall score | age 30-49 | 93.3 | 4.7 | 2.0 | 0.848 | 0.476, 1.510 | 0.575 |
|  | age 50-64 | 86.3 | 10.8 | 2.9 | 0.418 | 0.250, 0.698 | < 0.001 |
|  | age 65-74 | 83.9 | 13.2 | 2.9 | 0.343 | 0.199, 0.592 | < 0.001 |
|  | age ≥ 75 | 82.9 | 10.5 | 6.6 | 0.355 | 0.187, 0.675 | 0.002 |
|  | female | 87.8 | 9.3 | 2.9 |  |  |  |
|  | male | 89.4 | 8.6 | 2.0 | 1.176 | 0.890, 1.554 | 0.254 |
|  | low education | 78.8 | 16.2 | 5.0 |  |  |  |
|  | medium education | 88.9 | 8.4 | 2.7 | 1.925 | 1.374, 2.698 | < 0.001 |
|  | high education | 91.6 | 6.7 | 1.7 | 2.632 | 1.975, 3.508 | < 0.001 |
|  |  |  |  |  |  |  |  |
| **Alcohol use** | total | 88.1 | 5.5 | 6.4 |  |  |  |
|  | age 18-29 | 92.2 | 4.5 | 3.3 |  |  |  |
| Overall score | age 30-49 | 89.6 | 4.0 | 6.4 | 0.709 | 0.431, 1.165 | 0.174 |
| Lancet score | age 50-64 | 87.7 | 5.8 | 6.5 | 0.611 | 0.385, 0.969 | 0.036 |
| Lancet lifestyle subscore | age 65-74 | 86.6 | 6.3 | 7.1 | 0.589 | 0.356, 0.973 | 0.039 |
|  | age ≥ 75 | 80.1 | 10.7 | 9.2 | 0.415 | 0.231, 0.746 | 0.003 |
|  | female | 89.0 | 4.4 | 6.6 |  |  |  |
|  | male | 86.1 | 8.0 | 5.9 | 0.772 | 0.596, 1.000 | 0.050 |
|  | low education | 81.2 | 8.3 | 10.5 |  |  |  |
|  | medium education | 90.0 | 5.1 | 4.9 | 1.960 | 1.381, 2.781 | < 0.001 |
|  | high education | 89.9 | 4.6 | 5.5 | 1.979 | 1.489, 2.629 | < 0.001 |
|  |  |  |  |  |  |  |  |
| **Mental activity** | total | 87.9 | 9.6 | 2.5 |  |  |  |
|  | age 18-29 | 91.9 | 4.8 | 3.3 |  |  |  |
| Overall score | age 30-49 | 91.2 | 5.5 | 3.3 | 0.839 | 0.507, 1.387 | 0.493 |
| Lancet lifestyle subscore | age 50-64 | 87.3 | 10.8 | 1.9 | 0.620 | 0.393, 0.978 | 0.040 |
|  | age 65-74 | 83.3 | 14.5 | 2.2 | 0.468 | 0.288, 0.763 | 0.002 |
|  | age ≥ 75 | 86.2 | 11.8 | 2.0 | 0.669 | 0.359, 1.244 | 0.204 |
|  | female | 88.8 | 9.1 | 2.1 |  |  |  |
|  | male | 86.2 | 10.4 | 3.4 | 0.776 | 0.598, 1.007 | 0.057 |
|  | low education | 78.1 | 16.5 | 5.4 |  |  |  |
|  | medium education | 88.4 | 10.4 | 1.2 | 1.978 | 1.421, 2.754 | < 0.001 |
|  | high education | 91.4 | 6.7 | 1.9 | 2.914 | 2.192, 3.872 | < 0.001 |
|  |  |  |  |  |  |  |  |
| **Healthy diet** | total | 84.9 | 11.4 | 3.7 |  |  |  |
|  | age 18-29 | 83.4 | 14.6 | 2.0 |  |  |  |
| Overall score | age 30-49 | 87.7 | 8.4 | 3.9 | 1.277 | 0.860, 1.898 | 0.226 |
|  | age 50-64 | 84.6 | 11.5 | 3.9 | 1.123 | 0.787, 1.604 | 0.523 |
|  | age 65-74 | 83.5 | 12.7 | 3.8 | 1.040 | 0.695, 1.555 | 0.849 |
|  | age ≥ 75 | 84.0 | 10.7 | 5.3 | 1.221 | 0.719, 2.072 | 0.460 |
|  | female | 86.3 | 10.3 | 3.4 |  |  |  |
|  | male | 82.1 | 13.4 | 4.5 | 0.716 | 0.566, 0.907 | 0.006 |
|  | low education | 72.6 | 21.8 | 5.6 |  |  |  |
|  | medium education | 83.6 | 13.5 | 2.9 | 1.955 | 1.456, 2.626 | < 0.001 |
|  | high education | 90.2 | 6.4 | 3.4 | 3.489 | 2.680, 4.543 | < 0.001 |
|  |  |  |  |  |  |  |  |
| **Parent with dementia** | total | 84.0 | 9.4 | 6.6 |  |  |  |
|  | age 18-29 | 91.5 | 5.6 | 2.9 |  |  |  |
| Overall score | age 30-49 | 90.2 | 5.1 | 4.7 | 0.797 | 0.491, 1.296 | 0.361 |
|  | age 50-64 | 83.7 | 10.3 | 6.0 | 0.463 | 0.299, 0.718 | < 0.001 |
|  | age 65-74 | 75.6 | 13.4 | 11.0 | 0.287 | 0.181, 0.455 | < 0.001 |
|  | age ≥ 75 | 69.9 | 18.3 | 11.8 | 0.236 | 0.138, 0.403 | < 0.001 |
|  | female | 85.5 | 8.8 | 5.7 |  |  |  |
|  | male | 80.2 | 11.2 | 8.6 | 0.711 | 0.566, 0.894 | 0.711 |
|  | low education | 78.3 | 13.2 | 8.5 |  |  |  |
|  | medium education | 84.7 | 8.9 | 6.4 | 1.305 | 0.954, 1.785 | 0.096 |
|  | high education | 85.7 | 8.4 | 5.9 | 1.497 | 1.150, 1.949 | 0.003 |
|  |  |  |  |  |  |  |  |
| **Social activity** | total | 81.4 | 15.6 | 3.0 |  |  |  |
|  | age 18-29 | 81.1 | 17.3 | 1.6 |  |  |  |
| Overall score | age 30-49 | 81.8 | 14.3 | 3.9 | 0.886 | 0.613, 1.283 | 0.523 |
| Lancet score | age 50-64 | 81.2 | 15.8 | 3.0 | 1.012 | 0.719, 1.425 | 0.945 |
| Lancet lifestyle subscore | age 65-74 | 80.4 | 16.3 | 3.3 | 1.016 | 0.689, 1.498 | 0.937 |
|  | age ≥ 75 | 84.5 | 14.2 | 1.3 | 1.915 | 1.104, 3.323 | 0.021 |
|  | female | 83.6 | 13.7 | 2.7 |  |  |  |
|  | male | 76.5 | 19.8 | 3.7 | 0.583 | 0.468, 0.727 | < 0.001 |
|  | low education | 63.8 | 31.6 | 4.6 |  |  |  |
|  | medium education | 79.7 | 18.5 | 1.8 | 2.342 | 1.779, 3.084 | < 0.001 |
|  | high education | 88.7 | 8.4 | 2.9 | 4.829 | 3.761, 6.200 | < 0.001 |
|  |  |  |  |  |  |  |  |
| **Depression** | total | 78.3 | 9.7 | 12.0 |  |  |  |
|  | age 18-29 | 76.2 | 14.0 | 9.8 |  |  |  |
| Overall score | age 30-49 | 73.8 | 11.3 | 14.9 | 0.816 | 0.590, 1.128 | 0.218 |
| Lancet score | age 50-64 | 79.0 | 8.2 | 12.8 | 1.146 | 0.842, 1.559 | 0.387 |
| Lancet medical subscore | age 65-74 | 84.4 | 6.7 | 8.9 | 1.761 | 1.215, 2.553 | 0.003 |
|  | age ≥ 75 | 78.3 | 13.1 | 8.6 | 1.251 | 0.781, 2.004 | 0.351 |
|  | female | 80.6 | 7.8 | 11.6 |  |  |  |
|  | male | 72.9 | 14.3 | 12.8 | 0.612 | 0.500, 0.750 | < 0.001 |
|  | low education | 73.5 | 13.7 | 12.8 |  |  |  |
|  | medium education | 78.7 | 10.6 | 10.7 | 1.471 | 1.109, 1.950 | 0.007 |
|  | high education | 79.9 | 7.9 | 12.2 | 1.581 | 1.244, 2.011 | < 0.001 |
|  |  |  |  |  |  |  |  |
| **Physical activity** | total | 77.7 | 19.5 | 2.8 |  |  |  |
|  | age 18-29 | 76.2 | 23.1 | 0.7 |  |  |  |
| Overall score | age 30-49 | 81.8 | 14.9 | 3.3 | 1.266 | 0.898, 1.786 | 0.178 |
| Lancet score | age 50-64 | 77.0 | 19.9 | 3.1 | 1.086 | 0.795, 1.482 | 0.604 |
| Lancet lifestyle subscore | age 65-74 | 73.2 | 24.1 | 2.7 | 0.905 | 0.639, 1.283 | 0.575 |
|  | age ≥ 75 | 82.1 | 13.9 | 4.0 | 1.860 | 1.128, 3.065 | 0.015 |
|  | female | 79.6 | 17.6 | 2.8 |  |  |  |
|  | male | 73.1 | 24.0 | 2.9 | 0.663 | 0.539, 0.814 | < 0.001 |
|  | low education | 60.6 | 33.8 | 5.6 |  |  |  |
|  | medium education | 76.3 | 22.0 | 1.7 | 2.112 | 1.623, 2.749 | < 0.001 |
|  | high education | 84.6 | 13.1 | 2.3 | 3.673 | 2.911, 4.636 | < 0.001 |
|  |  |  |  |  |  |  |  |
| **Stress** | total | 72.0 | 13.7 | 14.3 |  |  |  |
|  | age 18-29 | 86.7 | 9.7 | 3.6 |  |  |  |
| Overall score | age 30-49 | 74.3 | 10.8 | 14.9 | 0.431 | 0.295, 0.629 | < 0.001 |
|  | age 50-64 | 68.0 | 14.7 | 17.3 | 0.326 | 0.228, 0.465 | < 0.001 |
|  | age 65-74 | 69.4 | 16.5 | 14.1 | 0.361 | 0.245, 0.532 | < 0.001 |
|  | age ≥ 75 | 66.7 | 18.6 | 14.7 | 0.320 | 0.200, 0.514 | < 0.001 |
|  | female | 73.4 | 12.2 | 14.4 |  |  |  |
|  | male | 68.6 | 17.3 | 14.1 | 0.765 | 0.633, 0.926 | 0.006 |
|  | low education | 65.2 | 19.1 | 15.7 |  |  |  |
|  | medium education | 74.7 | 14.1 | 11.2 | 1.463 | 1.124, 1.903 | 0.005 |
|  | high education | 73.2 | 11.7 | 15.1 | 1.415 | 1.135, 1.763 | 0.002 |
|  |  |  |  |  |  |  |  |
| **Smoking** | total | 71.9 | 15.6 | 12.5 |  |  |  |
|  | age 18-29 | 72.6 | 18.3 | 9.1 |  |  |  |
| Overall score | age 30-49 | 75.4 | 12.5 | 12.1 | 1.133 | 0.827, 1.551 | 0.437 |
| Lancet score | age 50-64 | 72.4 | 14.7 | 12.9 | 1.007 | 0.754, 1.345 | 0.962 |
| Lancet lifestyle subscore | age 65-74 | 68.8 | 16.6 | 14.6 | 0.835 | 0.604, 1.155 | 0.277 |
|  | age ≥ 75 | 62.5 | 25.7 | 11.8 | 0.675 | 0.445, 1.024 | 0.064 |
|  | female | 72.1 | 15.1 | 12.8 |  |  |  |
|  | male | 71.4 | 17.0 | 11.6 | 0.989 | 0.816, 1.198 | 0.989 |
|  | low education | 64.6 | 20.6 | 14.8 |  |  |  |
|  | medium education | 71.6 | 16.5 | 11.9 | 1.337 | 1.035, 1.728 | 0.026 |
|  | high education | 74.7 | 13.4 | 11.9 | 1.560 | 1.253, 1.944 | < 0.001 |
|  |  |  |  |  |  |  |  |
| **Traumatic brain injury** | total | 70.9 | 12.5 | 16.6 |  |  |  |
|  | age 18-29 | 82.1 | 11.4 | 6.5 |  |  |  |
| Overall score | age 30-49 | 72.1 | 11.9 | 16.0 | 0.586 | 0.416, 0.826 | 0.002 |
| Lancet score | age 50-64 | 68.4 | 13.8 | 17.8 | 0.481 | 0.349, 0.665 | < 0.001 |
| Lancet medical subscore | age 65-74 | 67.4 | 12.7 | 19.9 | 0.443 | 0.310, 0.632 | < 0.001 |
|  | age ≥ 75 | 70.0 | 9.1 | 20.9 | 0.496 | 0.314, 0.783 | 0.003 |
|  | female | 69.4 | 12.8 | 17.8 |  |  |  |
|  | male | 74.4 | 11.9 | 13.7 | 1.284 | 1.057, 1.561 | 0.012 |
|  | low education | 70.6 | 11.6 | 17.8 |  |  |  |
|  | medium education | 72.2 | 12.4 | 15.4 | 1.013 | 0.777, 1.319 | 0.926 |
|  | high education | 70.5 | 12.9 | 16.6 | 0.957 | 0.764, 1.198 | 0.702 |
|  |  |  |  |  |  |  |  |
| **Sleep disturbances** | total | 70.2 | 12.8 | 17.0 |  |  |  |
|  | age 18-29 | 80.1 | 12.1 | 7.8 |  |  |  |
| Overall score | age 30-49 | 71.7 | 12.1 | 16.2 | 0.615 | 0.440, 0.860 | 0.004 |
|  | age 50-64 | 67.6 | 13.5 | 18.9 | 0.533 | 0.390, 0.728 | < 0.001 |
|  | age 65-74 | 66.9 | 12.3 | 20.8 | 0.525 | 0.371, 0.742 | < 0.001 |
|  | age ≥ 75 | 70.9 | 14.5 | 14.6 | 0.666 | 0.424, 1.046 | 0.077 |
|  | female | 70.4 | 13.1 | 16.5 |  |  |  |
|  | male | 69.8 | 12.1 | 18.1 | 0.950 | 0.786, 1.148 | 0.593 |
|  | low education | 59.5 | 18.0 | 22.5 |  |  |  |
|  | medium education | 72.4 | 12.0 | 15.6 | 1.722 | 1.335, 2.222 | < 0.001 |
|  | high education | 73.1 | 11.4 | 15.5 | 1.834 | 1.479, 2.273 | < 0.001 |
|  |  |  |  |  |  |  |  |
| **Hearing loss** | total | 64.0 | 21.4 | 14.6 |  |  |  |
|  | age 18-29 | 50.2 | 36.1 | 13.7 |  |  |  |
| Overall score | age 30-49 | 55.8 | 27.2 | 17.0 | 1.135 | 0.856, 1.504 | 0.378 |
| Lancet score | age 50-64 | 67.5 | 17.4 | 15.1 | 2.084 | 1.597, 2.719 | < 0.001 |
| Lancet medical subscore | age 65-74 | 72.7 | 14.1 | 13.2 | 2.770 | 2.024, 3.791 | < 0.001 |
|  | age ≥ 75 | 77.1 | 15.7 | 7.2 | 4.184 | 2.666, 6.566 | < 0.001 |
|  | female | 66.4 | 18.9 | 14.7 |  |  |  |
|  | male | 58.7 | 27.1 | 14.2 | 0.656 | 0.546, 0.788 | < 0.001 |
|  | low education | 54.7 | 30.0 | 15.3 |  |  |  |
|  | medium education | 59.9 | 23.5 | 16.6 | 1.502 | 1.172, 1.926 | 0.001 |
|  | high education | 69.3 | 17.3 | 13.4 | 2.285 | 1.840, 2.839 | < 0.001 |
|  |  |  |  |  |  |  |  |
| **Diabetes** | total | 56.9 | 16.9 | 26.2 |  |  |  |
|  | age 18-29 | 49.6 | 26.6 | 23.8 |  |  |  |
| Overall score | age 30-49 | 58.6 | 15.5 | 25.9 | 1.389 | 1.051, 1.834 | 0.021 |
| Lancet score | age 50-64 | 57.5 | 16.1 | 26.4 | 1.370 | 1.058, 1.774 | 0.017 |
| Lancet medical subscore | age 65-74 | 59.4 | 13.2 | 27.4 | 1.474 | 1.097, 1.981 | 0.10 |
|  | age ≥ 75 | 54.3 | 17.9 | 27.8 | 1.252 | 0.846, 1.855 | 0.262 |
|  | female | 57.8 | 16.0 | 26.2 |  |  |  |
|  | male | 54.8 | 19.1 | 26.1 | 0.893 | 0.750, 1.062 | 0.200 |
|  | low education | 52.5 | 21.5 | 26.0 |  |  |  |
|  | medium education | 54.2 | 20.0 | 25.8 | 1.122 | 0.883, 1.426 | 0.345 |
|  | high education | 59.7 | 13.9 | 26.4 | 1.366 | 1.112, 1.678 | 0.003 |
|  |  |  |  |  |  |  |  |
| **High blood pressure** | total | 49.6 | 26.3 | 24.1 |  |  |  |
|  | age 18-29 | 47.3 | 34.1 | 18.6 |  |  |  |
| Overall score | age 30-49 | 49.1 | 26.7 | 24.2 | 1.045 | 0.791, 1.379 | 0.759 |
| Lancet score | age 50-64 | 50.1 | 23.5 | 26.4 | 1.140 | 0.880, 1.476 | 0.321 |
| Lancet medical subscore | age 65-74 | 51.0 | 27.5 | 21.5 | 1.143 | 0.852, 1.535 | 0.373 |
|  | age ≥ 75 | 48.6 | 24.1 | 27.3 | 1.094 | 0.738, 1.622 | 0.655 |
|  | female | 48.7 | 25.9 | 25.4 |  |  |  |
|  | male | 51.6 | 27.4 | 21.0 | 1.111 | 0.935, 1.321 | 0.233 |
|  | low education | 42.2 | 33.0 | 24.8 |  |  |  |
|  | medium education | 45.5 | 29.5 | 25.0 | 1.158 | 0.911, 1.472 | 0.232 |
|  | high education | 53.9 | 22.7 | 23.4 | 1.607 | 1.307, 1.975 | < 0.001 |
|  |  |  |  |  |  |  |  |
| **Elevated cholesterol** | total | 48.7 | 20.3 | 31.0 |  |  |  |
|  | age 18-29 | 49.2 | 25.4 | 25.4 |  |  |  |
| Overall score | age 30-49 | 46.5 | 21.0 | 32.5 | 0.846 | 0.641, 1.117 | 0.239 |
| Lancet score | age 50-64 | 49.8 | 19.3 | 30.9 | 1.021 | 0.788, 1.322 | 0.874 |
| Lancet medical subscore | age 65-74 | 48.5 | 17.7 | 33.8 | 0.983 | 0.732, 1.319 | 0.907 |
|  | age ≥ 75 | 49.0 | 21.2 | 29.8 | 1.069 | 0.721, 1.584 | 0.741 |
|  | female | 50.3 | 18.5 | 31.2 |  |  |  |
|  | male | 45.0 | 24.4 | 30.6 | 0.791 | 0.665, 0.941 | 0.008 |
|  | low education | 41.8 | 25.4 | 32.8 |  |  |  |
|  | medium education | 47.0 | 22.6 | 30.4 | 1.260 | 0.990, 1.603 | 0.060 |
|  | high education | 51.9 | 17.4 | 30.7 | 1.570 | 1.276, 1.931 | < 0.001 |
|  |  |  |  |  |  |  |  |
| **Obesity** | total | 48.5 | 30.7 | 20.8 |  |  |  |
|  | age 18-29 | 48.2 | 37.1 | 14.7 |  |  |  |
| Overall score | age 30-49 | 46.3 | 31.9 | 21.8 | 0.878 | 0.664, 1.160 | 0.358 |
| Lancet score | age 50-64 | 49.9 | 29.2 | 20.9 | 1.078 | 0.832, 1.397 | 0.571 |
| Lancet lifestyle subscore | age 65-74 | 48.8 | 27.3 | 23.9 | 1.011 | 0.752, 1.358 | 0.943 |
|  | age ≥ 75 | 48.0 | 32.7 | 19.3 | 1.059 | 0.714, 1.572 | 0.776 |
|  | female | 48.3 | 31.0 | 20.7 |  |  |  |
|  | male | 48.9 | 30.0 | 21.1 | 1.000 | 0.841, 1.190 | 1.000 |
|  | low education | 39.1 | 38.4 | 22.5 |  |  |  |
|  | medium education | 44.2 | 35.0 | 20.8 | 1.249 | 0.980, 1.591 | 0.072 |
|  | high education | 53.7 | 26.1 | 20.2 | 1.852 | 1.503, 2.280 | < 0.001 |
|  |  |  |  |  |  |  |  |
| **Vision loss** | total | 44.9 | 32.8 | 22.3 |  |  |  |
|  | age 18-29 | 37.8 | 45.6 | 16.6 |  |  |  |
| Overall score | age 30-49 | 40.1 | 35.7 | 24.2 | 1.047 | 0.787, 1.393 | 0.753 |
| Lancet score | age 50-64 | 46.0 | 29.8 | 24.2 | 1.432 | 1.098, 1.867 | 0.008 |
| Lancet medical subscore | age 65-74 | 51.3 | 28.6 | 20.1 | 1.858 | 1.374, 2.511 | < 0.001 |
|  | age ≥ 75 | 49.7 | 29.8 | 20.5 | 1.854 | 1.243, 2.765 | 0.002 |
|  | female | 45.9 | 30.4 | 23.7 |  |  |  |
|  | male | 42.4 | 38.3 | 19.3 | 0.819 | 0.687, 0.977 | 0.027 |
|  | low education | 35.1 | 41.1 | 23.8 |  |  |  |
|  | medium education | 43.5 | 33.0 | 23.5 | 1.581 | 1.234, 2.025 | < 0.001 |
|  | high education | 49.0 | 29.7 | 21.3 | 1.955 | 1.579, 2.420 | < 0.001 |
|  |  |  |  |  |  |  |  |
| **Education** | total | 41.3 | 51.4 | 7.3 |  |  |  |
|  | age 18-29 | 34.2 | 59.3 | 6.5 |  |  |  |
| Overall score | age 30-49 | 37.3 | 56.3 | 6.4 | 1.036 | 0.772, 1.390 | 0.813 |
| Lancet score | age 50-64 | 45.1 | 47.1 | 7.8 | 1.612 | 1.227, 2.117 | < 0.001 |
|  | age 65-74 | 44.0 | 47.5 | 8.5 | 1.513 | 1.111, 2.060 | 0.009 |
|  | age ≥ 75 | 39.3 | 54.8 | 5.9 | 1.480 | 0.980, 2.235 | 0.063 |
|  | female | 42.2 | 50.8 | 7.0 |  |  |  |
|  | male | 39.5 | 52.5 | 8.0 | 0.855 | 0.714, 1.023 | 0.087 |
|  | low education | 28.6 | 62.7 | 8.7 |  |  |  |
|  | medium education | 34.1 | 59.0 | 6.9 | 1.368 | 1.056, 1.773 | 0.018 |
|  | high education | 49.1 | 43.9 | 7.0 | 2.591 | 2.075, 3.235 | < 0.001 |
|  |  |  |  |  |  |  |  |
| **Air pollution** | total | 39.1 | 31.4 | 29.5 |  |  |  |
|  | age 18-29 | 44.6 | 38.8 | 16.6 |  |  |  |
| Overall score | age 30-49 | 39.0 | 30.6 | 30.4 | 0.812 | 0.614, 1.074 | 0.145 |
| Lancet score | age 50-64 | 38.1 | 31.7 | 30.2 | 0.772 | 0.595, 1.001 | 0.051 |
| Lancet lifestyle subscore | age 65-74 | 38.4 | 26.9 | 34.7 | 0.762 | 0.565, 1.026 | 0.073 |
|  | age ≥ 75 | 38.4 | 29.1 | 32.5 | 0.737 | 0.494, 1.101 | 0.136 |
|  | female | 37.6 | 30.5 | 31.9 |  |  |  |
|  | male | 42.8 | 33.2 | 24.0 | 1.238 | 1.039, 1.475 | 0.017 |
|  | low education | 39.8 | 32.8 | 27.4 |  |  |  |
|  | medium education | 39.1 | 31.0 | 29.9 | 0.952 | 0.745, 1.215 | 0.691 |
|  | high education | 39.0 | 30.8 | 30.2 | 0.950 | 0.771, 1.172 | 0.635 |
|  |  |  |  |  |  |  |  |
| **Heart disease** | total | 36.5 | 31.1 | 32.4 |  |  |  |
|  | age 18-29 | 46.6 | 31.6 | 21.8 |  |  |  |
| Overall score | age 30-49 | 40.7 | 28.7 | 30.6 | 0.767 | 0.581, 1.014 | 0.063 |
|  | age 50-64 | 34.9 | 31.4 | 33.7 | 0.616 | 0.474, 0.799 | < 0.001 |
|  | age 65-74 | 30.2 | 33.6 | 36.2 | 0.497 | 0.367, 0.674 | < 0.001 |
|  | age ≥ 75 | 27.2 | 32.4 | 40.4 | 0.444 | 0.290, 0.680 | < 0.001 |
|  | female | 37.1 | 29.2 | 33.7 |  |  |  |
|  | male | 35.0 | 35.7 | 29.3 | 0.929 | 0.775, 1.113 | 0.425 |
|  | low education | 30.7 | 35.2 | 34.1 |  |  |  |
|  | medium education | 36.3 | 32.5 | 31.2 | 1.172 | 0.909, 1.513 | 0.221 |
|  | high education | 38.7 | 29.1 | 32.2 | 1.341 | 1.077, 1.669 | 0.009 |
|  |  |  |  |  |  |  |  |
| **Sex** | total | 28.8 | 43.2 | 28.0 |  |  |  |
|  | age 18-29 | 51.8 | 30.9 | 17.3 |  |  |  |
| Overall score | age 30-49 | 40.3 | 34.8 | 24.9 | 0.567 | 0.427, 0.754 | < 0.001 |
|  | age 50-64 | 23.4 | 45.3 | 31.3 | 0.275 | 0.209, 0.362 | < 0.001 |
|  | age 65-74 | 14.1 | 55.0 | 30.9 | 0.154 | 0.108, 0.220 | < 0.001 |
|  | age ≥ 75 | 15.2 | 51.7 | 33.1 | 0.191 | 0.116, 0.316 | < 0.001 |
|  | female | 31.6 | 40.0 | 28.4 |  |  |  |
|  | male | 22.6 | 50.1 | 27.3 | 0.637 | 0.517, 0.785 | < 0.001 |
|  | low education | 15.9 | 54.1 | 30.0 |  |  |  |
|  | medium education | 30.0 | 44.5 | 25.5 | 1.834 | 1.352, 2.487 | < 0.001 |
|  | high education | 33.1 | 38.5 | 28.4 | 2.326 | 1.776, 3.048 | < 0.001 |
|  |  |  |  |  |  |  |  |
| **Excessive computer gaming** | total | 26.6 | 48.7 | 24.7 |  |  |  |
|  | age 18-29 | 24.7 | 58.7 | 16.6 |  |  |  |
| sham item | age 30-49 | 23.9 | 53.5 | 22.6 | 0.944 | 0.685, 1.303 | 0.727 |
|  | age 50-64 | 27.5 | 44.4 | 28.1 | 1.132 | 0.842, 1.522 | 0.413 |
|  | age 65-74 | 27.5 | 44.8 | 27.7 | 1.170 | 0.837, 1.637 | 0.358 |
|  | age ≥ 75 | 32.5 | 49.0 | 18.5 | 1.439 | 0.934, 2.216 | 0.099 |
|  | female | 28.0 | 45.2 | 26.8 |  |  |  |
|  | male | 23.2 | 56.7 | 20.1 | 0.764 | 0.625, 0.934 | 0.008 |
|  | low education | 28.7 | 46.1 | 25.2 |  |  |  |
|  | medium education | 28.3 | 48.7 | 23.0 | 1.026 | 0.787, 1.337 | 0.850 |
|  | high education | 25.1 | 49.6 | 25.3 | 0.879 | 0.699, 1.106 | 0.270 |
|  |  |  |  |  |  |  |  |
| **Aluminum** | total | 22.2 | 28.6 | 49.2 |  |  |  |
|  | age 18-29 | 17.9 | 41.4 | 40.7 |  |  |  |
| sham item | age 30-49 | 23.7 | 30.4 | 45.9 | 1.452 | 1.024, 2.058 | 0.036 |
|  | age 50-64 | 23.6 | 25.0 | 51.4 | 1.396 | 1.005, 1.938 | 0.046 |
|  | age 65-74 | 20.3 | 26.7 | 53.0 | 1.172 | 0.806, 1.706 | 0.406 |
|  | age ≥ 75 | 21.9 | 24.5 | 53.6 | 1.214 | 0.746, 1.976 | 0.436 |
|  | female | 22.7 | 25.5 | 51.8 |  |  |  |
|  | male | 21.0 | 35.9 | 43.1 | 0.935 | 0.758, 1.153 | 0.531 |
|  | low education | 25.0 | 24.5 | 50.5 |  |  |  |
|  | medium education | 23.7 | 26.6 | 49.7 | 0.934 | 0.707, 1.233 | 0.629 |
|  | high education | 20.6 | 30.9 | 48.5 | 0.772 | 0.606, 0.982 | 0.035 |
|  |  |  |  |  |  |  |  |
| **Kidney disease** | total | 21.9 | 34.6 | 43.5 |  |  |  |
|  | age 18-29 | 26.4 | 46.6 | 27.0 |  |  |  |
| Overall score | age 30-49 | 24.4 | 30.8 | 44.8 | 0.868 | 0.632, 1.191 | 0.381 |
|  | age 50-64 | 21.9 | 34.0 | 44.1 | 0.774 | 0.576, 1.042 | 0.091 |
|  | age 65-74 | 16.6 | 34.7 | 48.7 | 0.547 | 0.382, 0.784 | 0.001 |
|  | age ≥ 75 | 17.2 | 29.8 | 53.0 | 0.592 | 0.361, 0.973 | 0.039 |
|  | female | 22.5 | 32.8 | 44.7 |  |  |  |
|  | male | 20.0 | 39.2 | 40.8 | 0.881 | 0.712, 1.090 | 0.245 |
|  | low education | 19.1 | 43.5 | 37.4 |  |  |  |
|  | medium education | 21.1 | 35.4 | 43.5 | 1.032 | 0.766, 1.392 | 0.834 |
|  | high education | 23.2 | 31.0 | 45.8 | 1.211 | 0.938, 1.564 | 0.141 |
|  |  |  |  |  |  |  |  |
| **Mobile phone radiation** | total | 12.3 | 56.0 | 31.7 |  |  |  |
|  | age 18-29 | 20.2 | 59.3 | 20.5 |  |  |  |
| sham item | age 30-49 | 12.9 | 56.7 | 30.4 | 0.626 | 0.432, 0.907 | 0.013 |
|  | age 50-64 | 11.6 | 53.6 | 34.8 | 0.518 | 0.367, 0.730 | < 0.001 |
|  | age 65-74 | 8.3 | 57.9 | 33.8 | 0.380 | 0.244, 0.592 | < 0.001 |
|  | age ≥ 75 | 10.0 | 56.7 | 33.3 | 0.416 | 0.227, 0.766 | 0.005 |
|  | female | 12.8 | 52.3 | 34.9 |  |  |  |
|  | male | 11.1 | 64.5 | 24.4 | 0.866 | 0.660, 1.136 | 0.300 |
|  | low education | 13.4 | 46.8 | 39.8 |  |  |  |
|  | medium education | 17.4 | 52.6 | 30.0 | 1.214 | 0.867, 1.699 | 0.259 |
|  | high education | 9.6 | 60.9 | 29.5 | 0.644 | 0.470, 0.882 | 0.006 |

**Table S3: Mean knowledge scores**

| Score | Subgroup | Mean | SD | B | 95% CI | *p* |
| --- | --- | --- | --- | --- | --- | --- |
| **Overall score** | total | 14.34 | 4.82 |  |  |  |
| max. 23 | age 18-29 | 14.93 | 4.64 |  |  |  |
|  | age 30-49 | 14.61 | 4.72 | -0.548 | -1.197, 0.101 | 0.098 |
|  | age 50-64 | 14.26 | 4.94 | -0.621 | -1.225, -0.017 | 0.044 |
|  | age 65-74 | 13.94 | 4.68 | -0.862 | -1.550, -0.174 | 0.014 |
|  | age ≥ 75 | 13.71 | 5.12 | -0.657 | -1.575, 0.262 | 0.161 |
|  | female | 14.53 | 4.79 |  |  |  |
|  | male | 13.90 | 4.87 | -0.680 | -1.084, -0.276 | < 0.001 |
|  | low education | 12.42 | 5.27 |  |  |  |
|  | medium education | 14.16 | 4.60 | 1,640 | 1.081, 2.200 | < 0.001 |
|  | high education | 15.12 | 4.53 | 2,686 | 2.206, 3.165 | < 0.001 |
|  |  |  |  |  |  |  |
| **Lancet score** | total | 8.60 | 3.41 |  |  |  |
| max. 14 | age 18-29 | 8.41 | 3.35 |  |  |  |
|  | age 30-49 | 8.47 | 3.35 | -0.092 | -0.554, 0.369 | 0.694 |
|  | age 50-64 | 8.69 | 3.47 | 0.307 | -0.123, 0.736 | 0.161 |
|  | age 65-74 | 8.72 | 3.35 | 0.377 | -0.112, 0.867 | 0.130 |
|  | age ≥ 75 | 8.56 | 3.51 | 0.512 | -0.141, 1.165 | 0.124 |
|  | female | 8.70 | 3.39 |  |  |  |
|  | male | 8.37 | 3.45 | -0.400 | -0.687, -0.113 | 0.006 |
|  | low education | 7.45 | 3.68 |  |  |  |
|  | medium education | 8.36 | 3.28 | 0.970 | 0.573, 1.368 | < 0.001 |
|  | high education | 9.13 | 3.24 | 1,760 | 1.419, 2.101 | < 0.001 |
|  |  |  |  |  |  |  |
| **Lancet  medical subscore** | total | 4.13 | 2.04 |  |  |  |
| max. 7 | age 18-29 | 3.92 | 1.97 |  |  |  |
|  | age 30-49 | 3.96 | 2.05 | -0.034 | -0.313, 0.245 | 0.812 |
|  | age 50-64 | 4.18 | 2.06 | 0.263 | 0.003, 0.523 | 0.048 |
|  | age 65-74 | 4.33 | 2.04 | 0.441 | 0.145, 0.737 | 0.004 |
|  | age ≥ 75 | 4.24 | 2.05 | 0.465 | 0.070, 0.861 | 0.021 |
|  | female | 4.18 | 2.04 |  |  |  |
|  | male | 3.99 | 2.05 | -0.237 | -0.410, -0.063 | 0.008 |
|  | low education | 3.69 | 2.14 |  |  |  |
|  | medium education | 4.01 | 2.02 | 0.392 | 0.151, 0.633 | 0.001 |
|  | high education | 4.34 | 1.99 | 0.721 | 0.515, 0.928 | < 0.001 |
|  |  |  |  |  |  |  |
| **Lancet  lifestyle subscore** | total | 4.94 | 1.68 |  |  |  |
| max. 7 | age 18-29 | 5.07 | 1.73 |  |  |  |
|  | age 30-49 | 5.05 | 1.65 | -0.080 | -0.306, 0.146 | 0.489 |
|  | age 50-64 | 4.93 | 1.70 | -0.107 | -0.318, 0.103 | 0.319 |
|  | age 65-74 | 4.78 | 1.62 | -0.234 | -0.474, 0.006 | 0.056 |
|  | age ≥ 75 | 4.79 | 1.71 | -0.075 | -0.395, 0.245 | 0.646 |
|  | female | 4.98 | 1.65 |  |  |  |
|  | male | 4.84 | 1.75 | -0.153 | -0.294, -0.012 | 0.033 |
|  | low education | 4.26 | 1.92 |  |  |  |
|  | medium education | 4.89 | 1.63 | 0.606 | 0.410, 0.801 | < 0.001 |
|  | high education | 5.21 | 1.52 | 0.949 | 0.782, 1.116 | < 0.001 |
